# Effect of integrated management bundle on 1-year overall survival outcomes and perioperative outcomes in super elderly patients aged 90 and over with hip fracture: non-concurrent cohort study

**DOI:** 10.1186/s12891-022-05720-z

**Published:** 2022-08-15

**Authors:** Mingming Fu, Junfei Guo, Yaqian Zhang, Yuqi Zhao, Yingze Zhang, Zhiyong Hou, Zhiqian Wang

**Affiliations:** 1 Department of Geriatric Orthopedics, Third Hospital of Hebei Medical University, Hebei 050051 Shijiazhuang, People’s Republic of China; 2 Department of Orthopedic Surgery, Third Hospital of Hebei Medical University, Shijiazhuang, Hebei 050051 People’s Republic of China; 3 NHC Key Laboratory of Intelligent Orthopedic Equipment (The Third Hospital of Hebei Medical University), Hebei 050051 Shijiazhuang, People’s Republic of China; 4 Chinese Academy of Engineering, Beijing, 100088 People’s Republic of China

**Keywords:** Super elderly, Hip fracture, Management bundle, Perioperative complications, Survival

## Abstract

**Background:**

Due to concomitant factors like frailty and comorbidity, super elderly (≥90 years) patients with hip fracture differ from patients aged 65–89 years in perioperative complications and mortality. The integrated management bundle referred to bundled application of multiple clinical measures. The aim of this study was to analyze effect of integrated management bundle on 1-year overall survival and perioperative outcomes in super elderly patients with hip fracture, with multidisciplinary management group serving as the control group.

**Methods:**

In this retrospective cohort study, super elderly patients with hip fracture were included from Jan 2017 to Nov 2020. Patients were retrospectively divided to multidisciplinary management group and integrated management bundle group. The primary outcome was 1- year overall survival, and the secondary outcome was perioperative outcomes. Kaplan-Meier methods was used to compare survival probability. Multivariable Cox’s modeling was used to explain the effect of integrated bundle on 1-year overall survival adjusted for confounders. The perioperative outcomes including complications and in-hospital data of two groups were compared. The multivariable logistic regression was used to explain the effect of integrated bundle on the occurrence of perioperative complications adjusted for confounders. Prognostic factors related to survival was identified by multivariable Cox’s regression analysis.

**Results:**

Ninety-seven patients comprised multidisciplinary management group, and 83 comprised integrated management bundle group. The Kaplan–Meier plots showed that the survival probability of integrated management bundle group was significantly better than multidisciplinary management group (HR:0.435, 95%CI:0.207–0.914, *P* = 0.039). Multivariable analysis after adjustment for confounders showed a 42.8% lower incidence of mortality integrated management bundle group than multidisciplinary management group (HR:0.428, 95%CI:0.186–0.986, *P* = 0.046). Incidence of hypoproteinemia, and electrolyte disturbance in integrated management bundle group was significantly lower than multidisciplinary management group (all *P* < 0.05). In addition, significant reduction was observed in length of stay (*P* < 0.05) in integrated management bundle group. Multivariable logistic regression showed integrated management bundle was independent protective factor of hypoproteinemia, and electrolyte disturbance. mECM score ≥ 6 and ASA score > 2 were independent risk factors of overall survival (HR: 1.940, 95%CI: 1.067–3.525,*P* = 0.030; HR: 2.281, 95%CI: 1.113–4.678,*P* = 0.024).

**Conclusions:**

The integrated management bundle improved 1-year overall survival and played positive effects in improving perioperative outcomes. It might be a more suitable management modality for super elderly patients with hip fracture.

## Introduction

With increasing age, comorbidities, frailty, increased bone fragility, and loss of skeletal muscle mass increase the risk of falls and fractures [1]. As the most serious osteoporotic fracture, hip fracture can cause significant morbidity and mortality in older adults, persisting to be a burdensome public health problem. There are reports that the incidence of perioperative complications ranged from 20 to 75% in older adults with hip fracture [2]. No significant relationship is found between the occurrence of most perioperative complications and surgical procedures. Previous studies have confirmed that the incidence of nonsurgical complications is correlated with an increase in mortality and readmission of older adults with hip fracture [3, 4]. The super elderly adults aged 90 and over is a special population, and the number of whom is increasing. Due to concomitant factors such as frailty and comorbidity, the incidence of perioperative complications and mortality in hip fracture patients aged 90 and over may be higher than patients aged 65–89 years [5]. In face of this situation, effective interventions are needed to improve perioperative outcome and prognosis of hip fracture patients who are aged 90 and over.

Invernizzi M et al. recent work showed that multidisciplinary rehabilitation could improve performance and reduce disability in patients with hip fracture [6]. Trevisan C and colleagues suggest that better perioperative management and aggressive rehabilitation may further reduce mortality in elderly patients with hip fractures [7]. While there have been many studies about management of hip fractures patients, few studies have reported optimal management modality and outcomes of hip fracture patients who are aged 90 and over. More evidence is needed regarding the perioperative management of super elderly patients with hip fracture.

Like nursing bundle, the integrated management bundle referred to bundled application of multiple clinical measures. Every clinical measure that has proven effective. Several studies suggest that nursing bundle has a good clinical effect in reducing the risk of perioperative complications and improving prognosis [8]. Whether the integrated management bundle has the similar effects requires further investigation. Adverse outcomes after hip fracture surgery can be predicted by preoperative risk assessment. Some studies reported mECM may be favorable index for predicting major complications following hip fracture [9]. It remains to be further investigation whether the index will be applicable for special group such as super elderly patients with hip fracture.

The aim of this study was to analyze the effect of integrated management bundle on perioperative outcomes and early survival in super elderly patients with hip fracture, and provided more effective perioperative management modality for super elderly patients with hip fracture. It was hypothesized that this management modality could reduce perioperative complications and improve clinical outcomes.

## Methods

### Patients and groups

The retrospective cohort study was from a single Level I trauma center in China. Patients at Department of Geriatric Orthopedics, between Jan 2017 and Nov 2020 were retrospective reviewed. The Ethics Committee of the Third Hospital of Hebei Medical University approved study protocol (number 2021–087-1), and informed consent was exempted. Inclusion criteria was hip fracture patients aged 90 and over. Exclusion criteria were patients with pathological fractures, with non-surgical treatment, and with missing or incomplete data of follow up and perioperative period. Depending on the applied perioperative management modality, patients were retrospectively divided to multidisciplinary management group and integrated management bundle group. The primary outcome was 1-year overall survival. The secondary outcome was perioperative outcome, including the incidence of perioperative complications, total hospital costs, length of stay.

### Perioperative management

Unlike most hospitals, our hospital has a geriatric orthopaedic department, which consists of orthopedists, internists, rehabilitation specialists and trained nurses, and provides centralized management and 24/7 geriatric support [10]. The multidisciplinary geriatric fracture team attended ward rounds 7 days a week. These patients were evaluated by at least two orthopedic surgeons and an internist. Multidisciplinary management was applied in the first stage.

In the second stage, combined with the characteristics of patients and the unique medical system in my country, the existing management modality was simplified, optimized and integrated to form another modality named integrated management bundle, which was more consistent with the actual clinical situation and more practical. Specialists in orthopaedics, specialists in internal medicine with recognized expertise in geriatrics, anesthesiologists, rehabilitation physicians and specialized nursing staff were core members of the team. The internist performed comprehensive assessment of multisystem diseases based on a holistic view rather than only depending on clinical consultations. Multidisciplinary management group was in the first stage, while integrated management bundle group was in the second stage. The clinical measures of evaluation and education, nutritional support, respiratory management, volume management, blood management, thrombus management, pain management and sedation, tube management and preoperative protocol were different between two perioperative management modalities. The specific measures were described in Table 1 [10].

**Table 1:** Perioperative management measures

The multidisciplinary management	The integrated management bundle
1. The super elderly patients with hip fracture received monitor of electrocardiogram, mean arterial pressure and pulse oxygen saturation to ensure timely detection and treatment of complications [11, 12].	1. Evaluation and education: Electrocardiogram, mean arterial pressure, and pulse oxygen saturation were monitored in patients. A comprehensive geriatric examination was performed after admitted to identify potential risks and intervene in a timely manner [13]. The health care should be effectively preached, especially nutritional education.
2. Patient who recently had weight loss or a low body mass index on admission received assessment of nutritional status. Nutrition therapy was only available for a subset of patients [14].	2. Nutritional support: Patients were assessed for their nutritional status and performed nutritional treatment according to the specific situation. Use of probiotics and prokinetics was to prevent acute gastrointestinal dysfunction. Oral feeding was the main method. If food intake was insufficient, a nasogastric tube should be inserted to avoid electrolyte imbalance. Milk powder, protein powder, and enteral nutritional suspension were utilized as nutritional supplement [15, 16].
3. Patients with pulmonary infection or respiratory failure received oxygen treatment.	3. Respiratory management: Chest physiotherapy and breathing exercises were important, which included actively cough, accessary posture productive cough and turnover [17]. Low flow inhale oxygen and atomize were indispensable measures [10]. The patient received aerosol treatment of salbutamol sulfate, ipratropium bromide, and budesonide twice a day.
4. In order to prevent deep vein thrombosis, low molecular weight heparin and ankle pump exercise were administered according to the circumstances [18].	4. Volume management: The purpose of perioperative rehydration was to maintain fluid balance as much as possible [19].
5. To ameliorate pain, analgesics including opioid, nonsteroidal anti-inflammatory drug, or acetaminophen were given.	5. Blood management: In consideration of comorbidities and overall condition, patients were recommended to maintain an HGB level of at least 10 g per deciliter [18, 20–22].
6. Patients with suspected urinary retention received a single catheter. If urinary retention persisted, the catheter would remain in place for several days according to the circumstances [14].	6. Thrombus management: Actively take basic prevention, physical prevention, drug prevention and other measures to prevent lower extremity deep vein thrombosis [23, 24].
7. No food was allowed within 8 hours before the operation.	7. Pain management and sedation: Multimodal analgesia was suggested by clinical guidelines, included effective early analgesia, analgesic drugs and patient-controlled analgesia [16]. The mechanism of perioperative restlessness and delirium was complex [23, 24]. Identifying triggers and remove them were important, rather than rushing to medication.
	8. Tube management: Urinary retention was relieved by a single catheterization, and the second remained urethral catheter in place for 1 to 2 days [14].
	9. Carbohydrate-rich drinks and water might be consumed up to 2 hours before the operation. A normal diet was allowed 6 hours before the operation. The exceptions to this were patients experienced delayed gastric emptying and intestinal obstruction.

In the integrated management bundle group, interventions of evaluation and educationwere more intensive, tailored and idiographic than the multidisciplinary management group. In nutritional support, qualitative and quantitative measures was performed to assess nutritional status. The measures were more diversified and normative use than the multidisciplinary management group. In respiratory management, multiple treatment and prophylactic measures was valued. In the integrated management bundle group, volume management was clearly stated and normative use. As research and technology progresses, blood management was more valued. Preoperative correction of low hemoglobin levels can reduce 1-year all-cause mortality in hip fracture patients. In the integrated management bundle group, prophylactic measures of thrombus were diverse, and multimodal analgesia techniques were recommended. There is an increased emphasis on the prevention of disease in addition to its management.

### Data collection

All patient clinical data was collected from the patients’electronic medical records. Sex, age, Hb at admission, comorbidity, injury mechanism (low or high energy), injury place (indoor or outdoor), fracture type (femoral neck fracture or intertrochanteric fracture), fracture side (left or right), admission delay, surgery type (replacement or fixation), anesthesia type (general or regional), perioperative complications, total hospital cost, and length of stay were extracted. Follow-up started on the day the cohort was enrolled. The endpoint was the end of follow-up or the date of death, whichever came first. Patients in multidisciplinary management group were followed through Dec 2019. Patients in integrated management bundle group were followed non-concurrently. Each patient had at least 1 year of follow-up, and administrative censoring was performed at 1 year. The primary outcomes included 1-year overall survival. The secondary outcomes included the incidence of perioperative complications, total hospital costs, length of stay.

### Definition

Major perioperative complications included pulmonary infection, arrhythmia, anemia, deep vein thrombosis, heart failure and hypoalbuminemia. Pulmonary infection was defined as any pulmonary infection which was diagnosed by clinical and radiological evidence during a hospital stay. The arrhythmia detection method was ECG or ECG Monitoring System. Arrhythmia included sinus arrhythmia, premature atrial complex, premature ventricular complex, and atrial fibrillation or flutter. Anemia was defined according to the World Health Organization. Diagnosis of heart failure should be based on clinical signs, symptoms, prior cardiovascular history and further confirmed by appropriate additional investigations such as BNP, electrocardiogram, chest X-ray, and echocardiography. Electrolyte disturbances included hyponatremia (serum sodium< 135 mmol/L) and hypokalemia (Serum potassium< 3.5 mEq/L). Hypoalbuminemia was defined as serum albumin less than 30 g/L. The perioperative period was defined as admission for surgery, to their discharge.

### Potential prognostic factors

The potential prognostic factors comorbidities, ASA score, type of fracture, treatment factors (type of treatment, type of anesthesia, admission delay), and laboratory investigations (hemoglobin concentration at admission) [9, 25–28].

### Statistical analysis

Normally or approximately normally distributed variables were presented as mean and standard deviation (SD) and non-normal variables were summarized by median and interquartile range. Categorical variables were expressed as numbers and percentages. Differences between groups of continuous variables were compared using Student’s t-test or Mann-Whitney U test as appropriate, while the chi-square test or Fisher exact test for categorical variables [10]. Kaplan-Meier methods was used to compare survival, and log-rank test was used to assess any difference in survival. Multivariable Cox’s model was performed for the effect of integrated bundle on survival and determining the independent prognostic factors. The multivariable logistic regression to explain the effect of integrated bundle on the occurrence of perioperative complications. The correlation of modified Elixhauser’s Co-morbidity Measure (mECM) [29] and the number of perioperative complications were evaluated by Spearman’s correlation. The discrimination power of the predictors was evaluated using Harrell’s C concordance statistic (C-statistic). Statistical analyses were performed with SPSS V.26.0 and R statistical software. *P* < 0.05 was considered statistically significant.

## Results

### Patient characteristics

**Fig. 1:**
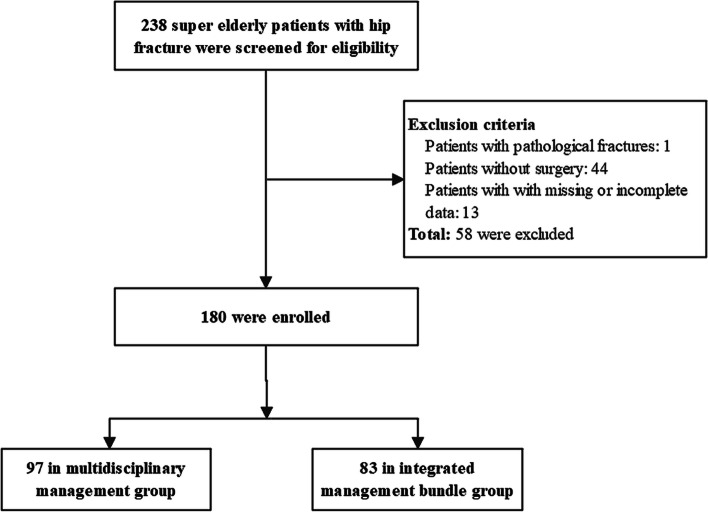
The flow diagram of the study

From Jan 2017 to Nov 2020, 180 patients were analyzed, of which 97 received multidisciplinary management and 83 underwent integrated management bundle (see Fig. 1). Most of patients were female (74.4%) and mean age of patients was 92.3 years (standard deviation 2.6). All patients were low-trauma hip fractures, and 163 (90.6%) of patients were injured indoors. The clinical characteristics of patients were shown in Table 2, and there was no significant difference between two groups (*P* > 0.05).

**Table 2:** Baseline characteristics of super elderly patients with hip fracture

	Total (***n*** = 180)	Multidisciplinary management group (***n*** = 97)	Integrated management bundle group (***n*** = 83)	***P***
**Sex,** ***n*** **(%)**				
**Male**	46(25.6%)	25 (25.8%)	21 (25.3%)	0.942
**Female**	134(74.4%)	72(74.2%)	62(74.7%)	
**Age, mean ± SD (years)**	92.3 ± 2.6	92.4 ± 2.9	92.1 ± 2.2	0.561
**Age group,** ***n*** **(%)**				
**90–94**	153 (85.0%)	80 (82.5%)	73 (88.0%)	0.305
**≥ 95**	27 (15.0%)	17 (17.5%)	10 (12.0%)	
**Hb at admission**	102.7 ± 16.6	102.2 ± 16.2	103.3 ± 17.2	0.611
**Hb group at admission,** ***n*** **(%)**				
**≥ 12 g/dl**	28 (15.6%)	16 (16.5%)	12 (14.5%)	0.769
**10-12 g/dl**	73 (40.6%)	36 (37.1%)	37 (44.6%)	
**8-10 g/dl**	64 (35.6%)	37 (38.1%)	27 (32.5%)	
**< 8 g/dl**	15 (8.3%)	8 (8.2%)	7 (8.4%)	
**ASA score,** ***n*** **(%)**				
**≤ 2**	54 (30.0%)	37 (38.1%)	25 (30.1%)	0.259
**> 2**	126 (70.0%)	60 (61.9%)	58 (69.9%)	
**mECM score,** ***n*** **(%)**				
**< 0**	39 (21.7%)	23 (23.7%)	19 (22.9%)	0.757
**0**	19 (10.6%)	8 (8.2%)	12 (14.5%)	
**1–5**	64 (35.6%)	35 (36.1%)	29 (34.9%)	
**6–13**	53 (29.4%)	29 (29.9%)	20 (24.1%)	
**≥ 14**	5 (2.8%)	2 (2.1%)	3 (3.6%)	
**Injury place,** ***n*** **(%)**				
**Indoor**	163 (90.6%)	89 (91.8%)	74 (89.2%)	0.553
**Outdoor**	17 (9.4%)	8 (8.2%)	9 (10.8%)	
**Fracture type,** ***n*** **(%)**				
**Femoral neck fracture**	61 (33.9%)	33 (34.0%)	28 (33.7%)	0.968
**Intertrochanteric fracture**	119 (66.1%)	64 (66.0%)	55 (66.3%)	
**Admission delay**	0.5 (0.2, 2.0)	0.4 (0.2, 2.0)	0.8 (0.2, 2.0)	0.498
**Surgical type,** ***n*** **(%)**				
**Replacement**	54 (30.0%)	29 (29.9%)	25 (30.1%)	0.974
**Fixation**	126(70.0%)	68 (70.1%)	58 (69.9%)	
**Anesthesia type,** ***n*** **(%)**				
**General**	82 (45.6%)	38 (39.2%)	44 (53.0%)	0.063
**Regional**	98 (54.4%)	59 (60.8%)	39 (47.0%)	

Values are presented as mean ± standard deviation, median (interquartile range), or number (percentage) as appropriate. *ASA* American Society of Anesthesiologists, *mECM* modified Elixhauser’s Comorbidity Measure

### 1-year overall survival analysis

**Fig. 2:**
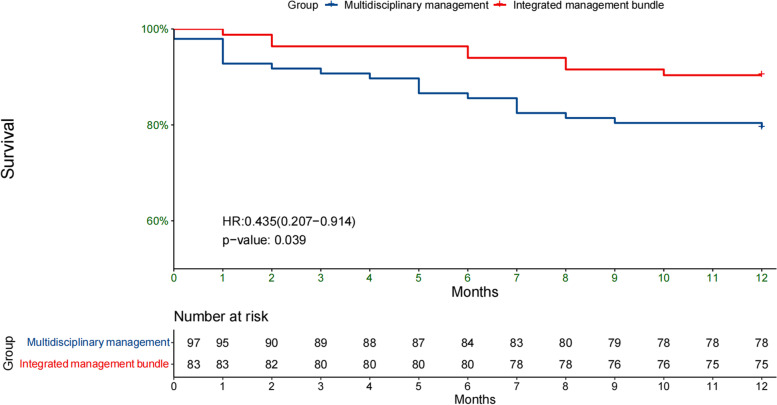
Kaplan - Meier curves of super elderly patients with hip fracture

1-year survival curves were compared by log-rank test and it was found that the integrated management package made improvement in survival (HR:0.435, 95%CI:0.207–0.914, *P* = 0.039). Kaplan-Meier survival curves of the two groups were shown in Fig. 2. Univariable analysis revealed that the patients in integrated management bundle group showed significant reduction in mortality rate compared to patients in multidisciplinary management (*P* = 0.047). All confounders had a variance inflation factor (VIF) value < 5. After multivariable analysis and adjusting for potential confounders including female, age ≥ 95 years, mECM score ≥ 6, ASA score > 2, intertrochanter fracture, admission delay ≥ 7 days, Hb at admission ≥ 10 g, regional anesthesia, a significant reduction in mortality rate of 42.8% was observed (*P* = 0.046) (Table 3).

**Table 3:** Univariable and multivariable analysis for the effect of integrated bundle on 1-year overall survival in hip fracture patients aged 90 and over

Characteristics	Univariable analysis		Multivariable analysis	
	HR (95% CI)	***P*** value	HR (95% CI)	***P*** value
**Sex**				
**Male**	Reference	0.932	Reference	0.620
**Female**	1.038 (0.441–2.441)		1.247 (0.520–2.990)	
**Age group**				
**90–94**	Reference	0.659	Reference	0.870
**≥ 95**	1.243 (0.473–3.270)		1.085 (0.407–2.890)	
**mECM score**				
**< 6**	Reference	0.026*	Reference	0.030*
**≥ 6**	2.321 (1.104–4.879)		2.401 (1.086–5.307)	
**ASA score**				
**≤ 2**	Reference	0.267	Reference	0.334
**> 2**	1.623(0.690–3.817)		1.558(0.634–3.826)	
**Fracture type**				
**Femoral neck**	Reference	0.518	Reference	0.376
**Intertrochanter**	0.779 (0.365–1.663)		0.691 (0.305–1.566)	
**Anesthesia type**				
**General**	Reference	0.831	Reference	0.664
**Regional**	0.922 (0.439–1.938)		0.846 (0.398–1.799)	
**Admission delay**				
**< 7 d**	Reference	0.552	Reference	0.669
**≥ 7 d**	0.646 (0.153–2.723)		0.720 (0.160–3.248)	
**Hb at admission**				
**< 10 g/dl**	Reference	0.886	Reference	0.533
**≥ 10 g/dl**	1056 (0.500–2.233)		0.765 (0.329–1.777)	
**Management modality**				
**Multidisciplinary management**	Reference	0.047*	Reference	0.046*
**Integrated management bundle**	0.435 (0.192–0.988)		0.428 (0.186–0.986)	

### Comparison of perioperative outcomes

The most common perioperative complications of super elderly hip fracture patients included anemia, hypoproteinemia and electrolyte disturbance. Compared with multidisciplinary management group, integrated management bundle group had a significantly reduction in incidence of hypoproteinemia and electrolyte disturbance (*P* = 0.031, and *P* = 0.045). There were no significant differences in the incidence of pulmonary infection, arrhythmia, heart failure, anemia, and deep vein thrombosis between two groups (All *P* > 0.05). The mean length of hospital stay among inpatients was 14.0 days in multidisciplinary management group. The mean length of hospital stay among inpatients was 12.6 days in integrated management bundle group. Length of stay was significantly lower in integrated management bundle group than multidisciplinary management group (*P* = 0.046). Detailed results were showed in Table 4. The multivariable logistic regression revealed that the integrated bundle was an independent protective factor of the occurrence of perioperative hypoalbuminemia and electrolyte disturbance adjusted for confounders (OR:0.476, 95%CI: 0.235–0.963, *P* = 0.039; OR:0.428, 95%CI: 0.216–0.846, *P* = 0.016). Detailed results were showed in Tables 5 and 6.

**Table 4:** Comparisons of perioperative complications and outcomes between two groups

Variables	Total (***n*** = 180)	Multidisciplinary management group (***n*** = 97)	Integrated management bundle group (***n*** = 83)	***P***
**Complications**				
**Pulmonary infection**				
**No**	110 (61.1%)	59 (60.8%)	51 (63.9%)	0.676
**Yes**	68 (37.8%)	38 (39.2%)	30 (36.1%)	
**Arrhythmia**				
**No**	114 (63.3%)	63 (64.9%)	51 (61.4%)	0.627
**Yes**	66 (36.7%)	34 (35.1%)	32 (38.6%)	
**Heart failure**				
**No**	85 (47.2%)	50 (51.5%)	35 (42.2%)	0.209
**Yes**	95 (52.8%)	47 (48.5%)	48 (57.8%)	
**Anemia**				
**No**	25 (13.9%)	12 (12.4%)	13 (15.7%)	0.524
**Yes**	155 (86.1%)	85 (87.6%)	70 (84.3%)	
**Deep vein thrombosis**				
**No**	95 (52.8%)	51 (52.6%)	44 (53.0%)	0.954
**Yes**	85 (47.2%)	46 (47.4%)	39 (47.0%)	
**Hypoalbuminemia**				
**No**	49 (27.2%)	20 (20.6%)	29 (34.9%)	0.031*
**Yes**	131 (72.8%)	77 (79.4%)	54 (65.1%)	
**Electrolyte disturbance**				
**No**	60 (33.3%)	26 (26.8%)	34 (41.0%)	0.045*
**Yes**	120 (66.7%)	71 (73.2%)	49 (59.0%)	
**In-hospital data**				
**Length of stay**	13.3 ± 4.8	14.0 ± 4.8	12.6 ± 4.7	0.046*
**Total hospital costs**	6.4 ± 1.8	6.4 ± 1.8	6.3 ± 1.8	0.799

Values are presented as mean ± standard deviation, or number (percentage) as appropriate. *P < 0.05, statistical significance

**Table 5:** Risk factors of perioperative hypoalbuminemia in hip fracture patients aged 90 and over analyzed by multivariable logistic regression

Variables	Wald z value	OR (95% CI)	***p*** value
**Integrated management bundle**	4.265	0.476 (0.235–0.963)	0.039
**Female**	4.166	0.387 (0.155–0.963)	0.041
**Age ≥ 95**	4.013	3.765 (1.029–13.777)	0.045
**mECM score ≥ 6**	0.533	0.741 (0.332–1.655)	0.465
**Intertrochanter fracture**	0.012	0.956 (0.433–2.112)	0.912
**ASA score > 2**	2.459	1.813(0.862–3.812)	0.117
**Admission delay ≥ 7 d**	2.431	2.972 (0.756–11.685)	0.119
**Hb at admission ≥ 10 g/dl**	2.454	0.531 (0.240–1.173)	0.117

**Table 6:** Risk factors of perioperative electrolyte disturbance in hip fracture patients aged 90 and over analyzed by multivariable logistic regression

Variables	Wald z value	OR (95% CI)	***p*** value
**Integrated management bundle**	5.952	0.428 (0.216–0.846)	0.016
**Female**	5.943	0.330 (0.136–0.805)	0.013
**Age ≥ 95**	7.124	0.286 (0.114–0.717)	0.010
**mECM ≥ 6**	0.229	0.812 (0.346–1.906)	0.949
**Intertrochanter fracture**	1.337	1.578 (0.728–3.421)	0.211
**ASA score > 2**	1.958	1.767 (0.796–3.920)	0.849
**Admission delay ≥ 7 d**	0.143	0.812 (0.275–2.392)	0.705
**Hb at admission ≥ 10 g/dl**	5.169	2.381(1.127–5.031)	0.031

### Prognostic factors for patients

Prognostic factors that might be associated with survival were analyzed using Cox proportional hazards model. In univariable Cox model, mECM score ≥ 6, and received integrated management bundle were the significant variables (*P* < 0.05). multivariable analysis showed mECM score ≥ 6 and ASA score > 2 were independent risk factors of overall survival (HR: 1.940, 95%CI: 1.067–3.525,*P* = 0.030; HR: 2.281, 95%CI: 1.113–4.678,*P* = 0.024). Detailed results were shown in Table 7. The C-index for the model was 0.654 (95%CI: 0.610–0.698).

**Table 7:** Cox proportional hazards regression model for overall survival

Characteristics	Univariable analysis		Multivariable analysis	
	HR (95% CI)	***P*** value	HR (95% CI)	***P*** value
**Sex**				
**Male**	Reference	0.133	Reference	0.463
**Female**	0.638 (0.355–1.146)		0.798 (0.438–1.457)	
**Age group**				
**90–94**	Reference	0.562	Reference	0.728
**≥ 95**	1.238 (0.601–2.548)		1.139 (0.549–2.362)	
**mECM**				
**< 6**	Reference	0.018*	Reference	0.030*
**≥ 6**	1.976 (1.125–3.469)		1.940 (1.067–3.525)	
**ASA score**				
**≤ 2**	Reference	0.035*	Reference	0.024*
**> 2**	2.060(1.053–4.029)		2.281 (1.113–4.678)	
**Fracture type**				
**Femoral neck**	Reference	0.148	Reference	0.193
**Intertrochanter**	0.657 (0.371–1.162)		0.660 (0.353–1.233)	
**Management modality**				
**Multidisciplinary management**	Reference	0.018*	Reference	0.007*
**Integrated management bundle**	0.487 (0.268–0.883)		0.430 (0.233–0.791)	
**Admission delay**				
**< 7d**	Reference	0.487	Reference	0.749
**≥ 7d**	0.696 (0.250–1.934)		0.839 (0.287–2.453)	
**Anesthesia type**				
**General**	Reference	0.558	Reference	0.530
**Regional**	1.183 (0.674–2.075)		1.200 (0.679–2.119)	
**Hb group at admission**				
**< 10 g**	Reference	0.353	Reference	0.602
**≥ 10 g**	1311(0.740–2.323)		0.843 (0.442–1.605)	

*Notes*: *P < 0.05, statistical significance. *ASA* American Society of Anesthesiologists, *mECM* modified Elixhauser’s Comorbidity Measure

### The correlations between mECM and the number of perioperative complications

Spearman correlation between mECM and the number of perioperative complications showed a weak positive correlation (*r* = 0.218, *P =* 0.003).

## Discussion

In our study, the characteristics and prognosis of super elderly patients aged 90 and over with hip fracture between two groups were analyzed. The application of integrated management bundle was found to be associated with better survival, lower incidence of complications including hypoproteinemia and electrolyte disturbance, and lower length of stay. mECM score ≥ 6 and ASA score > 2 were independent risk factors of overall survival. There was a weak positive correlation between mECM and the number of perioperative complications.

1-year postoperative mortality of hip fracture patients was found to be high. it will make sense to study how to reduce 1-year mortality rate. Some studies have reported some of management measures can reduce 1-year all-cause mortality in hip fracture patients. Our studies also have similar results [25]. From the results, we learned that the integrated management bundle made a significant survival benefit on the survival curve of super elderly hip fracture patients. Multivariable analysis after adjustment for confounders showed a 42.8% lower incidence of mortality integrated management bundle group than multidisciplinary management group. Nutritional counseling and education are important component of nutritional support. During hospitalization, we helped patients and their families form dietary perceptions appropriate for super elderly patients with hip fracture, which not only affected in-hospital outcomes, but also later in life. Blood management measures include correction of preoperative low hemoglobin levels. Worapaka Manosroi et.al reported preoperative correction of low hemoglobin levels can reduce 1-year all-cause mortality in hip fracture patients [25]. They believed that this was related to an increase of the oxygen-carrying capacity of blood. Anbar et al. [30] also demonstrated that nutritional support improved outcomes of elderly hip fracture patients. In addition to nutritional support, we also focus on volume management, respiratory management, etc. These bundled measures were the first step in developing a more comprehensive action. More studies were needed to better elucidate this effect.

In our study, the most common perioperative complications included anemia, hypoproteinemia and electrolyte disturbance. Multivariable logistic regression showed integrated management bundle was independent protective factor of hypoproteinemia, and electrolyte disturbance. These conditions were associated with traumatic stress response and aging. First, after hip fracture, elderly patients mount a severe stress response, including neuroendocrine response, immuno-inflammatory response and changes in the metabolic function of internal organs [31]. Specifically, Traumatic signals are relayed from injury site to central nervous system by a sensory afferent neuron. Next, hypothalamic-pituitary-adrenocortical axis are activated [32–36]. Therefore, stress hormones and catecholamines are released into the bloodstream. Increased anabolic hormone and decreased catabolic hormone put the body in a state of hypermetabolism, resulting in hyperglycemia, lipolysis, protein catabolism and hypoproteinemia [35, 36]. All the above factors and anorexia associated with the acute trauma and surgery induce low hemoglobin, low albumin, low sodium, low potassium and complications of various systems, causing serious hazards to the whole body. Second, aging is related to decline in physiological function of many organs, particularly leading to decline in intestinal digestion and absorption function, protein synthesis, and hemoglobin synthesis [37].

There is an abundance of data confirming the close relationship between albumin and inflammation. Previous studies have reported that albumin is considered a surrogate marker of inflammation status, participating in the systemic inflammatory response [38, 39]. Different from traditional inflammatory factors, albumin is a negative acute phase protein, and its level decreases with trauma and inflammation [40]. During the perioperative period, the increase of inflammatory factors promotes breakdown of albumin and reduce its synthesis. Therefore, perioperative hypoalbuminemia is attributed to several factors including albumin loss, protein catabolism, and inflammatory cytokines [41]. In addition, insufficient nutrient intake is a common problem in the perioperative period of elderly patients with hip fractures [42]. Studies show that nutritional support to patients at nutritional risk is advantageous. It could help to correct hypoproteinemia, and maintain the water, electrolyte and acid-base balance [30]. Our study results were consistent with these studies, providing new prognostic data.

In studies performed by Williams et al. [42], they revealed that early nutritional supplementation could significantly reduce hospital stay without increasing costs. Myint et al. [43] also found that oral nutritional supplementation reduced hospital stay and the number of infection episodes for elderly hip fracture. Despite specific interventions of nutritional support and age of subjects were not completely identical, the previous study demonstrated that nutritional support was benefit for hip fracture patients. However, Wyers et al. [44] reported intensive nutritional intervention after hip fracture did not improve LOS or clinical outcomes. In addition to the influence of several confounding factors, the results of the above study provided a new idea for us that the emphasis of only nutritional support might have been inadequate for elderly hip fracture patients. In our study, the bundled application of multiple measures was emphasized. The results showed that the incidence of hypoproteinemia, and electrolyte disturbance in integrated management bundle group was significantly lower than multidisciplinary management group. Length of stay was also significantly reduced. The reason was that the bundled application of multiple measures further reduced traumatic stress responses [10]. Referring to the specific mechanism described in the previous two paragraphs, it was not difficult to understand these results.

mECM score ≥ 6 and ASA score > 2 were independent risk factors of overall survival. Similar events have been reported in previous studies [9]. The mECM is possibly one of the best comorbidity indicators to predict major hip fracture complications [45]. In this study, mECM scores was calculated to determine the comorbidity burden at baseline. ASA score was considered as a physical status classification, using to assess the operative fitness status. The results also revealed that mECM was only weakly related to the number of perioperative complications, in special population namely patients aged 90 and over with hip fracture.

This study has distinct advantages. First, a comprehensive assessment of comorbidities and functional status was performed by using mECM scores and ASA grades. Second, we emphasized on the bundle application of management measures. Third, the study not only evaluated perioperative outcomes, but also early survival in super elderly patients aged 90 and over with hip fracture. The major limitation of this study is that it was a retrospective cohort study at a single center. The retrospective nature of the study implied a potential for inherent bias.

## Conclusions

The integrated management bundle yielded better perioperative outcome and early survival, which might be a more suitable management modality for super elderly hip fracture patients.

## Data Availability

The data that support the findings of this study are available from Zhiqian Wang but restrictions apply to the availability of these data, which were used under license for the current study, and so are not publicly available. Data are however available from the authors upon reasonable request and with permission of Zhiqian Wang.
